# Circadian variation pattern of sudden cardiac arrest occurred in Chinese community

**DOI:** 10.1136/openhrt-2024-002904

**Published:** 2024-10-16

**Authors:** Peng-Cheng Yao, Mo-Han Li, Mu Chen, Qian-Ji Che, Yu-Dong Fei, Guan-Lin Li, Jian Sun, Qun-Shan Wang, Yong-Bo Wu, Mei Yang, Ming-Zhe Zhao, Yu-Li Yang, Zhong-Xi Cai, Li Luo, Hong Wu, Yi-Gang Li

**Affiliations:** 1 Department of Cardiology, Xinhua Hospital Affiliated to Shanghai Jiao Tong University School of Medicine, Shanghai, China; 2 Shanghai Siwei Medical Co. Ltd, Shanghai, China; 3 School of Public Health, Fudan University, Shanghai, China; 4 Shanghai Municipal Health Commission, Shanghai, China; 5 Medical Information Telemonitoring Center, Shanghai Jiao Tong University School of Medicine, Shanghai, China

**Keywords:** Death, Sudden, Cardiac, Out-of-Hospital Cardiac Arrest, Arrhythmias, Cardiac

## Abstract

**Background:**

The circadian variation pattern of sudden cardiac arrest (SCA) occurred in Chinese community including both community healthcare centres and primary hospitals remains unknown. This study analysed the circadian variation of SCA in the Chinese community.

**Methods:**

Data between 2018 and 2022 from the remote ECG diagnosis system of Xinhua Hospital affiliated to Shanghai Jiao Tong University School of Medicine were analysed to examine the circadian rhythm of SCA, stratified by initial shockable (ventricular tachycardia or ventricular fibrillation) versus non-shockable (asystole or pulseless electrical activity) rhythm.

**Results:**

Among 10 210 cases of SCA, major cases (8736, 85.6%) were non-shockable and 1474 (14.4%) cases were shockable. The circadian rhythm of SCA was as follows: peak time was from 08:00 to 11:59 (30.1%), while deep valley was from 00:00 to 03:59 (7.5%). The proportions of events by non-shockable and shockable events were similar and both reached their peak from 08:00 to 11:59, with a percentage of 29.0% and 36.4%, respectively. Multivariable analysis showed that the relative risk of shockable compared with non-shockable arrests was lower between 00:00 and 03:59 (adjusted OR (aOR): 0.72, 95% CI: 0.54 to 0.97, p=0.028) and 04:00 to 07:59 (aOR: 0.60, 95% CI: 0.46 to 0.79, p<0.001), but higher between 08:00 and 11:59 (aOR: 1.34, 95% CI: 1.09 to 1.64, p=0.005).

**Conclusions:**

In Chinese community, there is a distinct circadian rhythm of SCA, regardless of initial rhythms. Our findings may be helpful in decision-making, in that more attention and manpower should be placed on the morning hours of first-aid and resuscitation management in Chinese community.

**WHAT IS ALREADY KNOWN ON THIS TOPIC**

**WHAT THIS STUDY ADDS**

- There is an obvious circadian rhythm in the occurrence of sudden cardiac arrest which may be helpful to guide first-aid and resuscitation in Chinese community, regardless of initial rhythms.

**HOW THIS STUDY MIGHT AFFECT RESEARCH, PRACTICE OR POLICY**

- The results could help guide first-aid and resuscitation, and thus benefit the community health.

## Introduction

 Sudden cardiac arrest (SCA), especially out-of-hospital cardiac arrest (OHCA), is the most time-critical and fatal medical emergency, while the treatment of in-hospital cardiac arrest (IHCA) is the most critical clinical challenge worldwide.^1 2^ Stratified by the initial cardiac rhythm, SCA is usually categorised into ventricular tachycardia or fibrillation (VT/VF) and asystole or pulseless electrical activity (asystole/PEA).^3^

Previous studies demonstrated that there is a circadian rhythm in SCA and both VT/VF and asystole/PEA are more likely to occur in the morning hours.^4^ OHCA incidence, regardless of initial rhythm, was reported to reach its peak in the morning.^5^ Research also showed that VT/VF outside the hospitals occurred more frequently in the morning^6^,^8^; asystole/PEA was seldom reported separately. Opinions diverge in terms of the circadian variation of IHCA. Results from Italy showed that the incidence of IHCA was the highest in the morning.^9^ However, data from the Get With the Guidelines-Resuscitation registry showed that there was no obvious circadian variation of IHCA throughout the day.^3^ The latest analysis of two UK databases shared a neutral standpoint in that the incidence of IHCA peaked in the morning, but the variation was absent in the intensive care unit.^10^

Most arrests occurred in communities, and community-based interventions for resuscitation appeared to be associated with improved survival following SCA.^11 12^ However, studies on SCA occurred in the community are insufficient. ECG plays a significant role in the monitoring of SCA, and a nationwide remote system is now established in China.^13 14^ Herein, we sought to determine the circadian variation of SCA in Chinese community, relying on the ECG data from this nationwide remote ECG diagnosis system in community healthcare centres and primary hospitals.

## Methods

### Design and study population

This is a non-randomised, cross-sectional study. Data from the remote ECG diagnosis system (platform) of Xinhua Hospital affiliated to Shanghai Jiao Tong University School of Medicine, which covered a total of 1600 medical institutions (community healthcare centres and primary hospitals) from 12 provinces in China with 24/7 availability, were analysed. ECGs with evidence of cardiac arrests, including VT/VF and asystole/PEA, were screened. Invalid data, including newborn and those missing critical demographics including age, sex or time of event, were excluded. For patients who underwent repeated ECG examinations, if there was evidence of VT/VF followed by asystole/PEA, the result of the ECG examination confirming VT/VF was retained; if VT/VF followed asystole/PEA, the latter was retained.

Study population included in this research came from different geographical areas of China, including the northwest (Qinghai, Xinjiang), the eastern (Jiangsu, Shanghai), the central (Hubei, Jiangxi), the southeast (Guangdong, Zhejiang) and the southwest (Chongqing, Guizhou, Tibet, Yunnan) and came from both economically developed areas (higher than the national per capita gross domestic product (GDP) level: Chongqing, Guangdong, Hubei, Jiangsu, Shanghai, Zhejiang) and economic less-developed areas (below the national per capita GDP level: Jiangxi, Qinghai, Xinjiang, Tibet, Yunnan, Guizhou).^15^

### Data collection and diagnosis

All the ECG data were collected by China Food and Drug Administration-certified ECG machines (EDAN SE-1201, MINDRAY BenenHeart R12, NIHON KOHDEN ECG-1250P and so on), which could contain the ECG data of the patients in resting state for 30 s and transmit automatically through the network after the collection. All ECG data, vector data in Digital Imaging and Communications in Medicine format, were encrypted and transmitted through the network. Besides, no data was missing in the transmission process, and invalid data were eliminated.

The demographic characteristics of objects include sex, age, location and the specific time of ECG examination. Convolutional neural network (CNN) was applied in ECG artificial intelligence diagnosis. The stability and accuracy of the machine-learning algorithm have been validated by the ECG platform. Take the algorithm for ventricular arrhythmias (VAs, including premature ventricular contraction and VT/VF) diagnosis as an example. A deep convolutional residual network (ResNet) was used to stack 15 residual units, and a total of 33 convolution layers were reached. Batch normalisation and dropout were performed on each layer, using a rectified linear unit as the activation function. Then, long short-term memory (LSTM) followed, aiming to summarise the information of each time step. The final output of LSTM was processed by the full connection layer, and Softmax function was used to output the two-dimensional prediction result: VAs or not. A total of 100 000 samples were collected as the training set, which contained 10 000 VAs and 90 000 non-VAs samples. Another 5000 samples, 500 of which were VAs, were used as the testing set. There was no intersection between the testing and the training sets. The machine learning algorithm was implemented via TensorFlow V.1.13.1. The hardware environment of the training process was NVIDIA GeForce RTX 2080 Ti and the operating system was Ubuntu V.18.04. In the training process, Adam (learning_rate=0.001) was used as the optimator. The maximum number of iterations was 50, and the loss-function was categorical_crossentropy. The training process would end when the loss-function could not be further reduced (accuracy was set to 1e-5) or the maximum number of iterations was reached. During the process of training, validation_split was set to 0.2 so that TensorFlow randomly divided 20% of the data as the validation set. After the training, the parameters of CNN were fixed and the data in the testing set were input for prediction. Similar algorithms were adopted in other ECG diagnoses.^15^

The preliminary artificial intelligence diagnosis was re-examined by two doctors. If the diagnosis of two doctors consisted of each other, or one of the doctors’ diagnosis and the artificial intelligence diagnosis were consistent, the consistent diagnosis would be reserved. If the three diagnosis were discorded, a third experienced physician was invited to review the ECG and the final consensus was achieved based on the opinions of at least two out of the four diagnosis opinions. The coincidence rate between two physicians on the same ECG diagnosis was 98% and that was 96% between physicians and machine learning algorithm.

### Statistical analysis

The relative proportion of SCA was examined over a 24-hour cycle in 4-hour intervals, and the relative proportion of events in each time interval was calculated as the total number of events during the 4-hour interval divided by the total number of events in 24 hours. Then, the relative proportion was further examined separately for patients with asystole/PEA and VT/VF in order to examine the relative risk of SCA occurred due to asystole/PEA versus VT/VF. Intergroup comparisons were made by χ^2^ test or Mann-Whitney U test, as appropriate. Univariate and multivariable analyses were also performed to examine the relative likelihood of the occurrence of asystole/PEA versus VT/VF in different time intervals, using the two-level hierarchical logistic regression model. The primary dependent variable was the initial rhythm. The independent variable was the time of occurrence in 4-hour intervals, and covariates included age, sex, weekend arrests, geographical locations of arrests, economic level (higher or lower than the national per capita GDP level) and categories of medical institutions (community healthcare centres or primary hospitals). The significance level threshold for the entry and exit of independent variables into the multivariable model was set at 0.05. Besides, the relative proportion of SCA was also examined hourly, weekly and monthly. Statistical analyses were performed by SPSS V.22.0 (IBM Software, Armonk, New York, USA). A two-sided p<0.05 was considered statistically significant.

## Results

ECG data, from the remote ECG diagnosis system of Xinhua Hospital affiliated to Shanghai Jiao Tong University School of Medicine between 1 January 2018 and 31 December 2022 were analysed. Initially, 5 394 504 ECG data were screened. Of the cases, there were 13 724 cases of SCA. After the exclusion of newborn and those missing critical demographics, the final sample included 10 210 cases (mean age: 68.5±21.4 years, 5494 men), among which 8736 (85.6%) cases were asystole/PEA and 1474 (14.4%) cases were VT/VF (online supplemental figure 1). The baseline characteristics are presented in table 1.

**Table 1:** Demographic, cardiac arrest and baseline characteristics

	Overall(n=10 210)	Asystole/PEA(n=8736)	VT/VF(n=1474)	P value
Age (years)	68.5±21.4	68.2±22.0	70.6±16.9	<0.001
Male	5494 (53.8)	4595 (52.6)	899 (61.0)	<0.001
Weekend arrest	2736 (26.8)	2348 (26.9)	388 (26.3)	0.657
Time interval of arrest				<0.001
00:00–03:59	763 (7.5)	678 (7.8)	85 (5.8)	
04:00–07:59	1114 (10.9)	1008 (11.5)	106 (7.2)	
08:00–11:59	3074 (30.1)	2537 (29.0)	537 (36.4)	
12:00–15:59	2690 (26.3)	2302 (26.4)	388 (26.3)	
16:00–19:59	1630 (16.0)	1409 (16.1)	221 (15.0)	
20:00–23:59	939 (9.2)	802 (9.2)	137 (9.3)	
Location of arrest				0.006
East	9342 (91.5)	7980 (91.3)	1362 (92.4)	
Central	61 (0.6)	53 (0.6)	8 (0.5)	
Southeast	164 (1.6)	132 (1.5)	32 (2.2)	
Southwest	305 (3.0)	281 (3.2)	24 (1.6)	
Northwest	338 (3.3)	290 (3.3)	48 (3.3)	
Economic developed areas	9538 (93.4)	8136 (93.4)	1402 (95.1)	0.015
Primary hospitals	6393 (62.6)	5457 (62.5)	936 (63.5)	0.447

Values are mean±SD or n (%).

PEA, pulseless electrical activity; VF, ventricular fibrillation; VT, ventricular tachycardia

**Figure 1:**
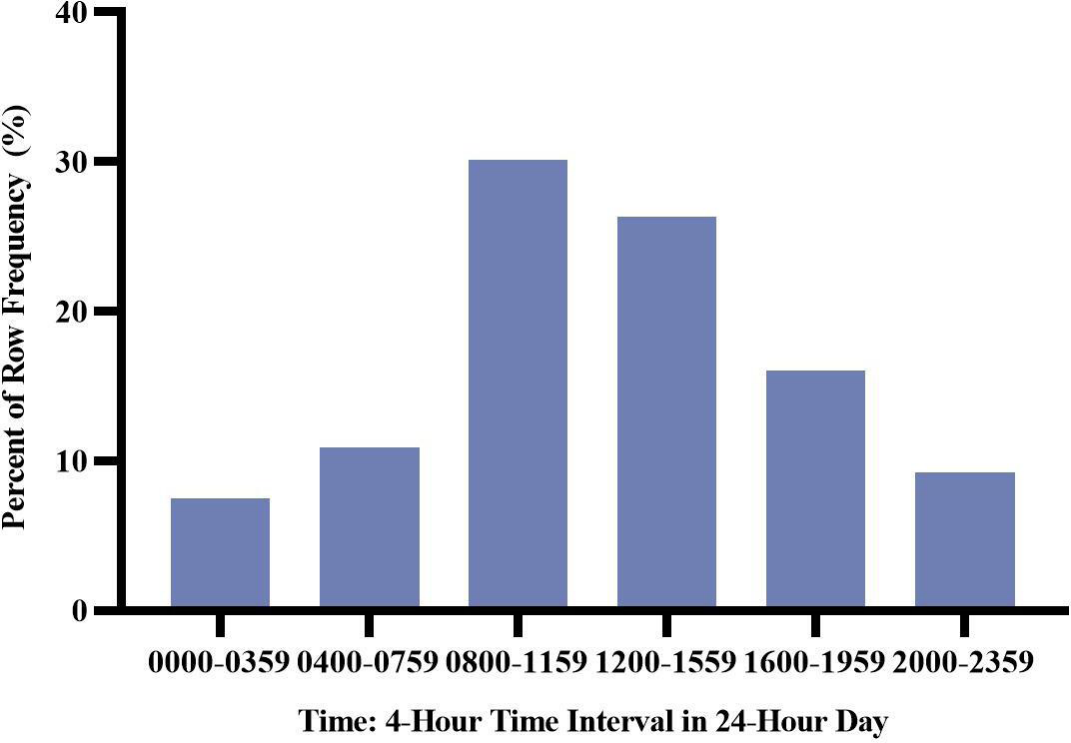
Proportion of sudden cardiac arrest events in Chinese community in 4-hour time interval over a 24-hour cycle.

**Figure 2:**
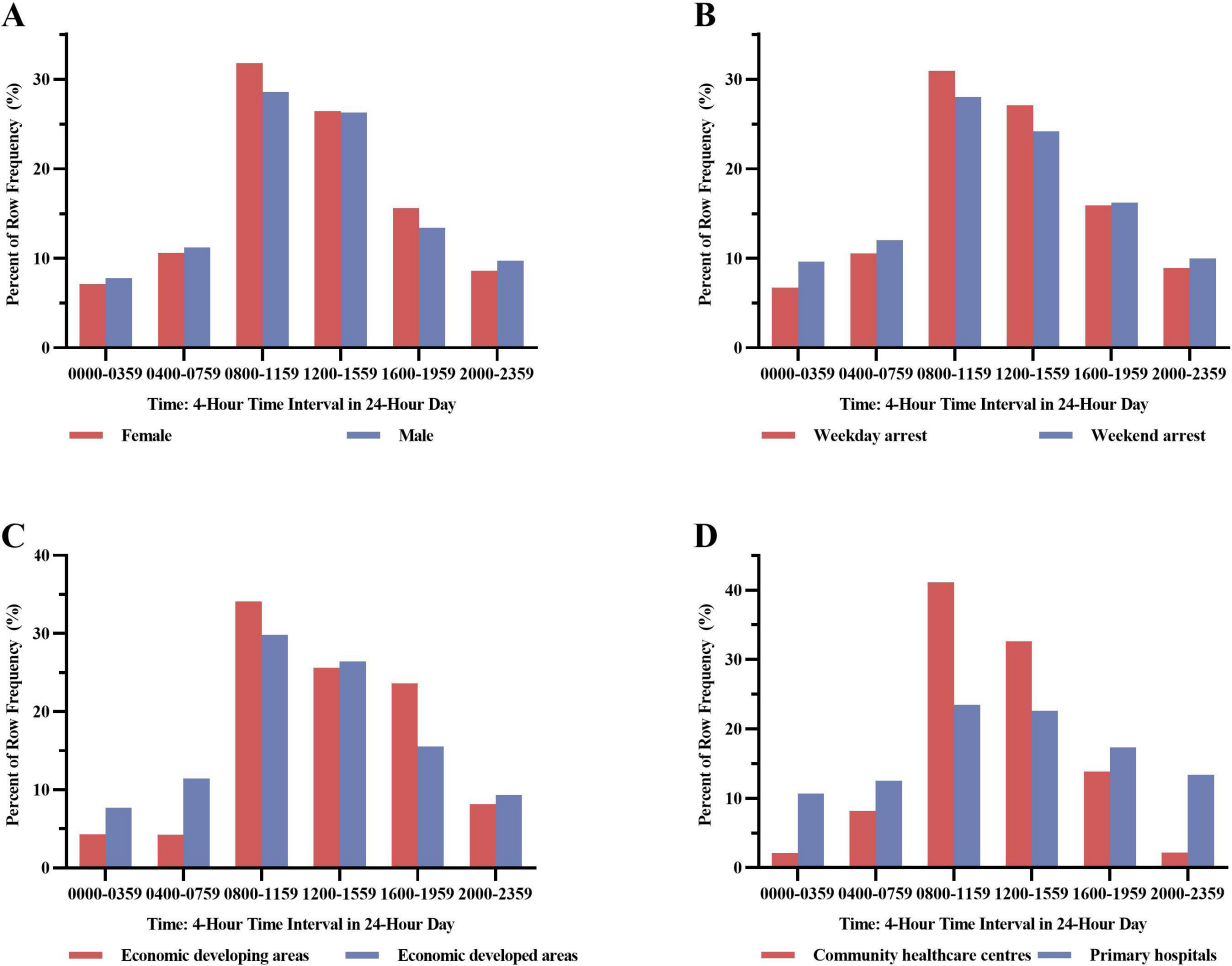
Proportion of sudden cardiac arrest events stratified by sex, time of arrests, economy and categories of medical institutions in 4-hour time interval over a 24-hour cycle.

**Figure 3:**
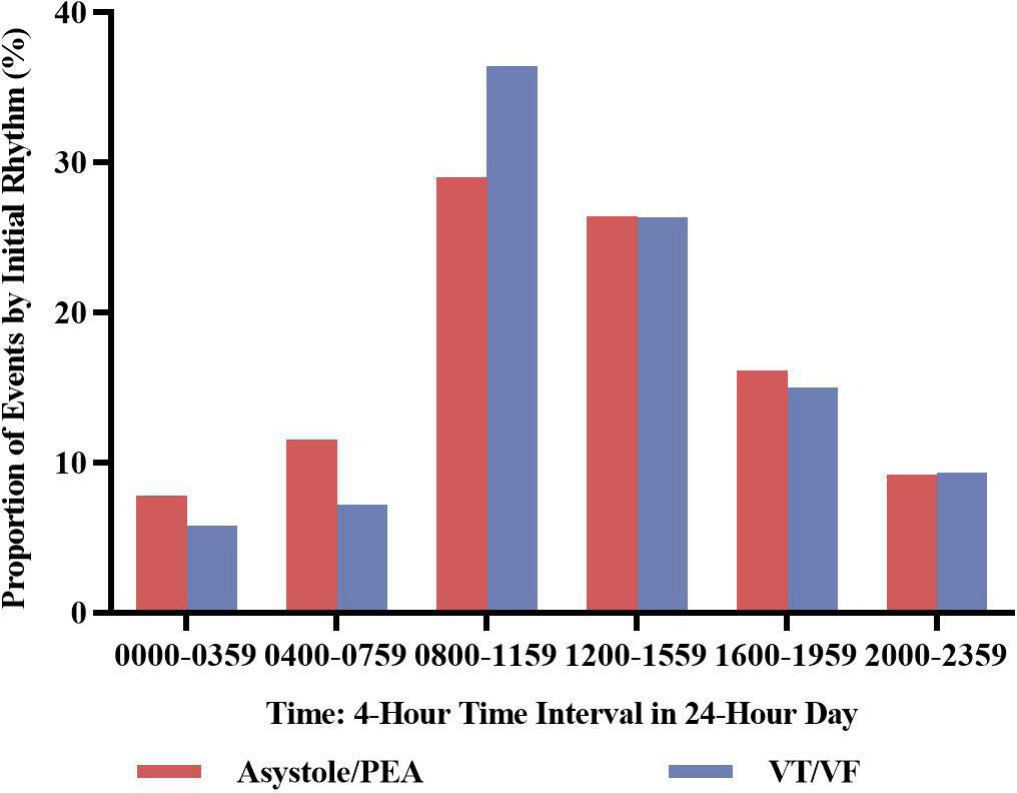
Circadian variation of sudden cardiac arrest occurred in different geographic locations in China. PEA, pulseless electrical activity; VF, ventricular fibrillation; VT, ventricular tachycardia.

Initially, the diurnal variation in the occurrence of SCA was analysed. As shown in figure 1, the occurrence of SCA reached its peak from 08:00 to 11:59 (30.1%), while fell into the deep valley from 00:00 to 03:59 (7.5%). Men and women nearly shared the same circadian trend in SCA (figure 2A), both peaking from 08:00 to 11:59. The difference was that the proportion of women was higher from 08:00 to 11:59 (31.8% vs 28.6%) and from 16:00 to 19:59 (15.6% vs 13.4%) than that of men, while slightly lower from 00:00 to 03:59 (7.1% vs 7.8%) and 20:00 to 23:59 (8.6% vs 9.7%). Also, the occurrence of SCA on the weekday or weekend (figure 2B) and in economically developed or developing areas (figure 2C) were almost similar to the overall trend of SCA. What is disparate was that, unlike the circadian variation of SCA in the community healthcare centres which was similar to the overall trend, the occurrence of SCA in the primary hospitals did not appear to show an obvious circadian variation (figure 2D). Besides, the circadian variation of SCA occurred in different geographical locations were analysed (figure 3). Specially, the pattern in the northwest of China showed two relative peaks, one from 08:00 to 11:59 (25.1%) and one from 16:00 to 19:59 (29.3%). Among the east, southeast, southwest and centre of China, where patterns of arrest showed a single peak from 08:00 to 11:59, the peak in the central part of Chinese community was the highest (50.8%).

Unadjusted univariate analysis demonstrated that there was statistically significant circadian variation in the relative proportion of both asystole/PEA and VT/VF (online supplemental figure 2). The proportions of events by asystole/PEA and VT/VF both reached their peak from 08:00 to 11:59, respectively, with a percentage of 29.0 and 36.4. SCA due to asystole/PEA, compared with VT/VF, was proportionally higher from 00:00 to 03:59 (7.8% vs 5.8%), 04:00 to 07:59 (11.5% vs 7.2%) and 16:00 to 19:59 (16.1% vs 15.0%). On the contrary, asystole/PEA was proportionally lower than VT/VF from 08:00 to 11:59 (29.0% vs 36.4%). The proportions of asystole/PEA and VT/VF were comparable from 12:00 to 15:59 (26.4% vs 26.3%) and 20:00 to 23:59 (9.2% vs 9.3%).

The circadian rhythm of the occurrence of SCA in 1-hour intervals was also researched (online supplemental figure 3). Unlike the trend shown in 4-hour intervals, the trend of both asystole/PEA and VT/VF in 1-hour intervals appeared to be double-peak. The first peak of the occurrence of asystole/PEA came from 10:00 to 10:59, and the second came from 15:00 to 15:59 with a similar proportion to the former one (8.6% vs 8.1%). The occurrence of VT/VF peaked first from 10:00 to 10:59, and peaked second from 14:00 to 14:59 with a percentage of 7.7, which was relatively lower than the former one (10.0%). Besides, the proportions of events by the two initial rhythms were similar from 18:00 to 01:59 the next day, except from 23:00 to 23:59. It is noted that the incidence of VT/VF was visibly higher than that of asystole/PEA (2.6% vs 2.0%).

The difference of SCA occurred in community healthcare centres and primary hospitals was further examined (online supplemental figure 4). It was shown that, compared with that in primary hospitals, a trend towards circadian patterns in community healthcare centres was more obvious, with a peak from 08:00 to 11:59 in the incidence of asystole/PEA and VT/VF. Moreover, compared with the incidence of asystole/PEA in community healthcare centres, that of VT/VF was more pronounced (38.6% vs 56.5%). What is more, the circadian variations of man or woman, weekday or weekend arrests and economic developing or developed areas were individually similar to the overall circadian trend in community healthcare centres. In primary hospitals, it was notable that the occurrence of SCA in economically developing areas was peaked from 16:00 to 19:59 with a percentage of 28.7. The circadian variations of the other categories were nearly the same as the overall trend in primary hospitals.

After adjustment for confounders (table 2), multivariable analysis showed that, compared with the reference interval of 20:00 to 23:59 when the incidence of VT/VF was comparable to that of asystole/PEA, relative risk of VT/VF compared with asystole/PEA was lower from 00:00 to 03:59 (adjusted OR (aOR): 0.72, 95% CI: 0.54 to 0.97, p=0.028) and from 04:00 to 07:59 (aOR: 0.60, 95% CI: 0.46 to 0.79, p<0.001), but higher from 08:00 to 11:59 (aOR: 1.34, 95% CI: 1.09 to 1.64, p=0.005). Also, after accounting for diurnal variations, the likelihood of VT/VF was higher with older age (aOR: 1.01, 95% CI: 1.01 to 1.01, p<0.001) and male sex (aOR: 1.47, 95% CI: 1.31 to 1.65, p<0.001), but lower in the southeast of China (aOR: 0.48, 95% CI: 0.29 to 0.81, p=0.006), compared with the likelihood of asystole/PEA.

**Table 2:** Multivariable analysis of the risk of cardiac arrest due to VT/VF compared with asystole/PEA

Characteristics	Univariate		Multivariable	
	OR (95% CI)	P value	OR (95% CI)	P value
Time interval of arrest				
00:00–03:59	0.73 (0.55 to 0.98)	0.036	0.72 (0.54 to 0.97)	0.028
04:00–07:59	0.62 (0.47 to 0.81)	<0.001	0.60 (0.46 to 0.79)	<0.001
08:00–11:59	1.24 (1.01 to 1.52)	0.039	1.34 (1.09 to 1.64)	0.005
12:00–15:59	0.99 (0.80 to 1.22)	0.901	1.07 (0.87 to 1.32)	0.536
16:00–19:59	0.92 (0.73 to 1.16)	0.467	0.97 (0.77 to 1.22)	0.788
20:00–23:59	Reference		Reference	
Age	1.01 (1.00 to 1.01)	<0.001	1.01 (1.01 to 1.01)	<0.001
Male	1.41 (1.26 to 1.58)	<0.001	1.47 (1.31 to 1.65)	<0.001
Weekend arrest	0.97 (0.86 to 1.10)	0.657		
Location of arrest				
East	1.03 (0.76 to 1.41)	0.846	0.40 (0.02 to 7.64)	0.544
Central	0.91 (0.41 to 2.04)	0.822	0.64 (0.03 to 12.37)	0.765
Southeast	1.47 (0.90 to 2.40)	0.129	0.48 (0.29 to 0.81)	0.006
Southwest	0.52 (0.31 to 0.87)	0.012	0.35 (0.02 to 6.61)	0.483
Northwest	Reference		Reference	
Economic developed areas	1.37 (1.06 to 1.76)	0.015	2.33 (0.13 to 43.52)	0.570
Primary hospitals	1.05 (0.93 to 1.17)	0.447		

PEA, pulseless electrical activity; VF, ventricular fibrillation; VT, ventricular tachycardia

The risk factors of SCA due to community healthcare centres compared with primary hospitals were also analysed for their discrepancies in circadian variation pattern (online supplemental table 1). Multivariable analysis showed that, compared with the reference interval of 04:00 to 07:59 when the incidence of SCA in community healthcare centres was comparable to that in primary hospitals, SCA risk in primary hospitals was lower from 08:00 to 11:59 (aOR: 0.41, 95% CI: 0.35 to 0.48, p<0.001) and 12:00 to 15:59 (aOR: 0.50, 95% CI: 0.42 to 0.58, p<0.001), and higher from 00:00 to 03:59 (aOR: 3.64, 95% CI: 2.77 to 4.78, p<0.001) and 20:00 to 23:59 (aOR: 4.49, 95% CI: 3.43 to 5.87, p<0.001) than that in community healthcare centres. Besides, compared with the reference area of the southeast where the incidences of SCA in community healthcare centres and primary hospitals were similar, SCA in the primary hospitals, compared with that in the community healthcare centres was less occurred in the east (aOR: 0.47, 95% CI: 0.33 to 0.68, p<0.001), central (aOR: 0.01, 95% CI: 0.00 to 0.03, p<0.001) and southwest (aOR: 0.00, 95% CI: 0.00 to 0.01, p<0.001) parts of China. The results also showed that the incidence of SCA in primary hospitals was higher with older age (aOR: 1.01, 95% CI: 1.00 to 1.01, p<0.001) and male sex (aOR: 1.25, 95% CI: 1.14 to 1.36, p<0.001), but lower in the economically developed areas (aOR: 0.02, 95% CI: 0.00 to 0.18, p=0.001).

Lastly, the weekly and seasonal variation of SCA was analysed. As shown in online supplemental figure 5, the weekly variation of the incidence of asystole/PEA and VT/VF both reached a nadir on Sunday and Monday, and the proportion oscillated around 16% from Tuesday to Saturday. When it came to the seasonal variation of SCA (online supplemental figure 6), the incidence of asystole/PEA flared up in December (12.4%), while that of VT/VF had a small peak in July (9.4%). The proportions of both initial rhythms in the rest months oscillated around 8%.

## Discussion

Unlike the characteristic of IHCA, SCA in Chinese community was more similar to OHCA, the occurrence of which appeared to peak in the morning hours (08:00–11:59). This pattern made no difference between men and women, weekday and weekend arrests or economically developed and developing areas, but differed when it came to community healthcare centres and primary hospitals, the latter of which seemed to have a much milder circadian variation when compared with that of the former one. The different circadian patterns in different geographical areas also made a difference: while the northwest presented a double-peak pattern, the other areas involved in this study presented a single-peak one. Besides, VT/VF SCA, due to its higher peak and lower nadir, had a more pronounced circadian variation than asystole/PEA SCA. Similar to the overall variation, the peaks of the two initial rhythms—both asystole/PEA and VT/VF—were milder in primary hospitals than in community healthcare centres. Furthermore, according to the multivariable analysis, the related risk of VT/VF compared with asystole/PEA was statistically higher from 08:00 to 11:59, with older age, or in men, while lower in from 00:00 to 07:59 or in the southeast.

The National Heart, Lung and Blood Institute Workshop reported that day/night rhythms in behaviours, the environment and endogenous circadian rhythms might result in a distinct 24-hour pattern in SCA occurrence.^16^ The risk factors of SCA also differed between the two initial rhythms. It was suggested that OHCA was inclined to be affected by adrenergic tone, blood pressure, heart rate and platelet aggregation, which were believed to have their own circadian rhythms.^17^ Also, the behaviour of being hospitalised itself could disrupt the circadian rhythm, and cause more OHCA, especially VT/VF.^3^ On the contrast, IHCA was possibly induced by medical therapies, especially those may have an effect on adrenergic tone, blood pressure and platelet activity.^3^ Besides, therapies in intensive care units could disrupt the circadian rhythms of patients, and thus led to more cardiac arrests.^18 19^

In our study, the total trend of SCA peaked at 08:00–11:59, appearing to be in accordance with OHCA. Instead of comparing OHCA and IHCA, we compared SCA in the community healthcare centres with that in the primary hospitals. It is demonstrated that SCA occurred in community healthcare centres was consistent with OHCA,^5^,^8^ while that in the primary hospitals was in keeping with the characteristics of IHCA in the Get With the Guidelines-Resuscitation registry,^3^ which did not show an obvious circadian variation. This difference suggested that community healthcare centres in China, though quite a part of them had the necessary medical facilities and expertise medical staff, cannot be comparable to general hospitals; primary hospitals, though still had quite a disparity from the superior ones, did not show a discrepancy in circadian variation of SCA even with hospitals in developed countries. This point was weighty that the primary hospitals in China were well-equipped and showed no weakness in medical quality from some points of view. Further, it is noteworthy that doctors working in community healthcare centres should pay more attention to the circadian variation of SCA for the reason that there might be more SCA events in the morning hours.

To the best of our knowledge, this is the first report exploring the circadian periodicity of SCA based on initial rhythms in Chinese community. This study may reflect a relatively lower incidence of shockable rhythm than the former research,^20 21^ probably for the reason that ECG was hardly done before defibrillation. When it came to the relative frequency of asystole/PEA to VT/VF, our research reported that asystole/PEA was proportionally higher in the late night/early morning hours (00:00–07:59). It was thought-provoking that this result was also reported in the American Heart Association’s investigation of IHCA.^3^ What is more, this study also declared that the relative proportion of VT/VF was higher from 08:00 to 11:59 than that of asystole/PEA. This point was different from the former investigation, which manifested a similar occurrence in 08:00–11:59 between asystole/PEA and VT/VF. However, according to the circadian rhythms of SCA in intensive or cardiac care units shown in the same study,^3^ the proportion of asystole/PEA was higher in 00:00–07:59, while lower in 08:00–11:59 when compared with VT/VF, which coincided with our results. The difference in the occurrence of non-shockable versus shockable events shown in our research suggested that, more attention and manpower should be placed on the morning hours on first-aid and resuscitation management in Chinese community. An observational study of cardiac arrests in Singapore demonstrated a definite geographical distribution pattern of cardiac arrests: OHCA occurrence was highest in the eastern and southern part of the country.^11^ In this study, due to the geographical feature of China, the southeast areas also showed a difference in the occurrence of asystole/PEA to VT/VF with the other areas when compared with the northwest. It may not be a coincidence that atmosphere temperature, patient characteristics, cardiac arrest circumstances could all be a reason.^11 22 23^ Besides, sex difference was observed in the occurrence of SCA in two initial rhythms: VT/VF was more frequently occurred in the male. This conclusion was also reported in the Amsterdam Resuscitation Study (ARREST).^24^

Furthermore, circaseptan and circannual periodicity of SCA were depicted. The rate of SCA from Tuesday to Saturday, regardless of initial rhythms, was roughly the same, while that on both Monday and Sunday was relatively lower. The low occurrence of SCA on Sunday was previously reported, but it remained controversial of the events rate on Monday: some may declare a maximum of events on Monday, while some argued a minimum.^25 26^ The relevance of naturally occurring rhythmic fluctuations in human physiology, and socially determined rhythms in human behaviours in different countries could be a reason of this discrepancy. We recorded a peak in events of asystole/PEA in December as well. Exposure to cold weather was considered to be one of the key factors. This information might alert physicians for SCA management in winter season.

## Limitations

This study has several potential limitations. First, a lack of information on treatment and comorbidity may limit the generalisability of our findings. In addition, even though ECG was proven to be an effective way of monitoring SCA in medical institutions, quick rhythm conversion between the initial non-shockable and shockable rhythm may make the results susceptible to bias and confounding.^13 27^ Finally, as an observational cross-sectional study, the influence of SCA occurred at different times on adverse events still remains elusive. A clinical follow-up should be conducted to address this issue if possible.

## Conclusions

There is an obvious circadian rhythm in the occurrence of SCA in Chinese community in that SCA occur mostly during morning hours (08:00 to 11:59), regardless of initial rhythms. Compared with SCA occurred in community healthcare centres, circadian variation in primary hospitals was milder. Age, sex, temporal and geographical difference could lead to the discrepancy in SCA events. Clinically, these findings may be helpful to guide first-aid and resuscitation in Chinese community.

## supplementary material

10.1136/openhrt-2024-002904 online supplemental file 1

## Data Availability

Data are available upon reasonable request.
