# Supplementary material for: Circadian variation pattern of sudden cardiac arrest occurred in Chinese community
Source: Open Heart. 2024 Oct 16;11(2):e002904. doi: 10.1136/openhrt-2024-002904 (PMC11487843; doi:10.1136/openhrt-2024-002904)
Supplement: online supplemental file 1 [file openhrt-11-2-s001.pdf]

Circadian variation pattern of sudden cardiac arrest occurred in Chinese community

Running tittle: Circadian rhythm and cardiac arrest

Peng-Cheng Yao[1, †], Mo-Han Li[1, †], Mu Chen[1, †], Qian-Ji Che[1], Yu-Dong Fei[1], Guan-Lin Li[1], Jian Sun[1], Qun-Shan Wang[1], Yong-Bo Wu[2], Mei-Yang[1], Ming-Zhe Zhao[1], Yu-Li Yang[1], Zhong-Xi Cai[2], Li Luo[3], Hong Wu[4], Yi-Gang Li[1, 5, *]

1 Department of Cardiology, Xinhua Hospital, School of Medicine, Shanghai Jiao Tong University, Shanghai, China.

2 Shanghai Siwei Medical Co. Ltd., Shanghai, China.

3 School of Public Health, Fudan University, Shanghai, China.

4 Shanghai Municipal Health Commission, Shanghai, China.

5 Medical Information Telemonitoring Center, School of Medicine, Shanghai Jiao Tong University, Shanghai, China.

† These authors contributed equally to this work.

*Correspondence: Yi-Gang Li, MD.

Department of Cardiology, Xinhua Hospital, School of Medicine, Shanghai Jiao Tong University

1665 Kongjiang Road, Shanghai 200092, China

Tel.: +86-21-25077265

E-mail: liyigang@xinhuamed.com.cn

Supplemental material

Supplemental Figure 1. Study flowchart.

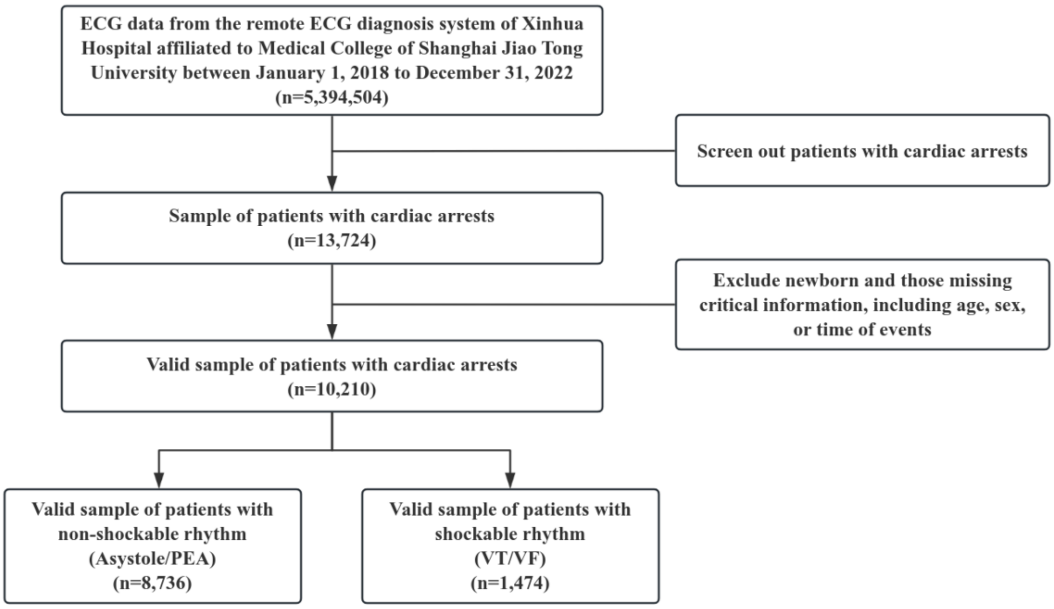

ECG = electrocardiogram; PEA = pulseless electrical activity; VF = ventricular fibrillation; VT = ventricular tachycardia.

Supplemental Figure 2. Proportion of sudden cardiac arrest events stratified by initial rhythms in 4-hour time interval over a 24-hour cycle.

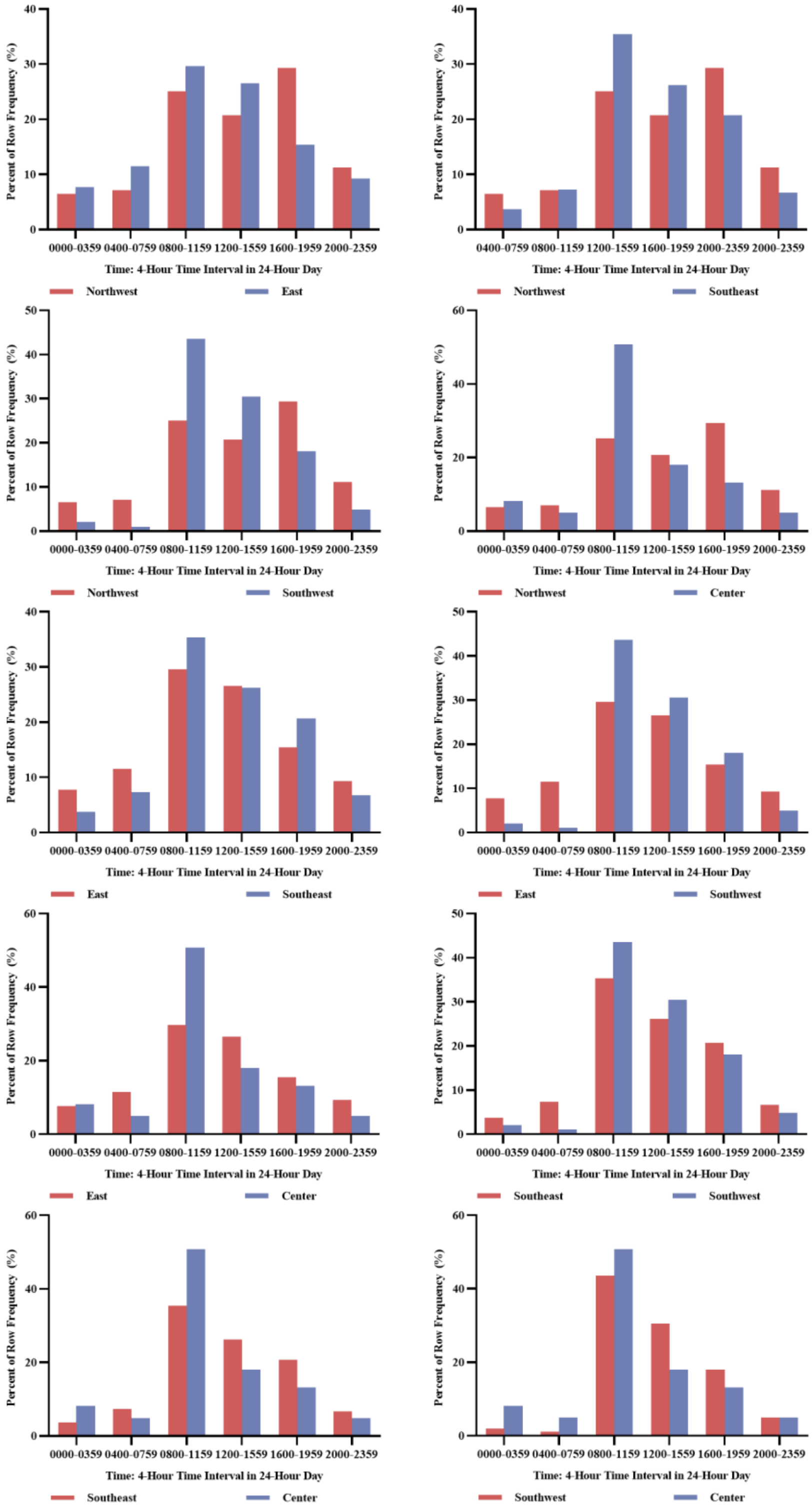

PEA = pulseless electrical activity; VF = ventricular fibrillation; VT = ventricular tachycardia.

Supplemental Figure 3. Circadian rhythm of the occurrence of sudden cardiac arrest in 1-hour intervals.

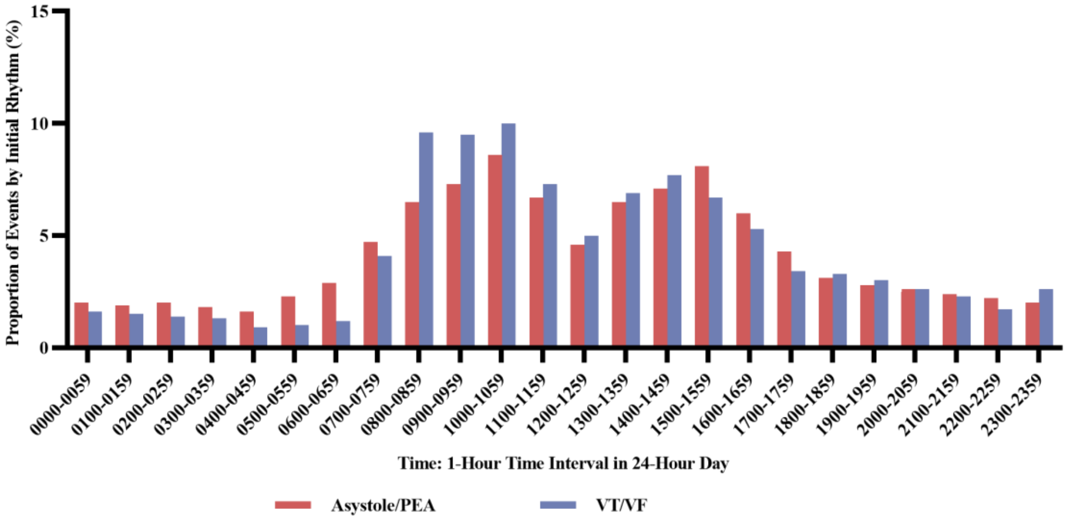

PEA = pulseless electrical activity; VF = ventricular fibrillation; VT = ventricular tachycardia.

Supplemental Figure 4. Sudden cardiac arrest occurred in community healthcare centers and primary hospitals.

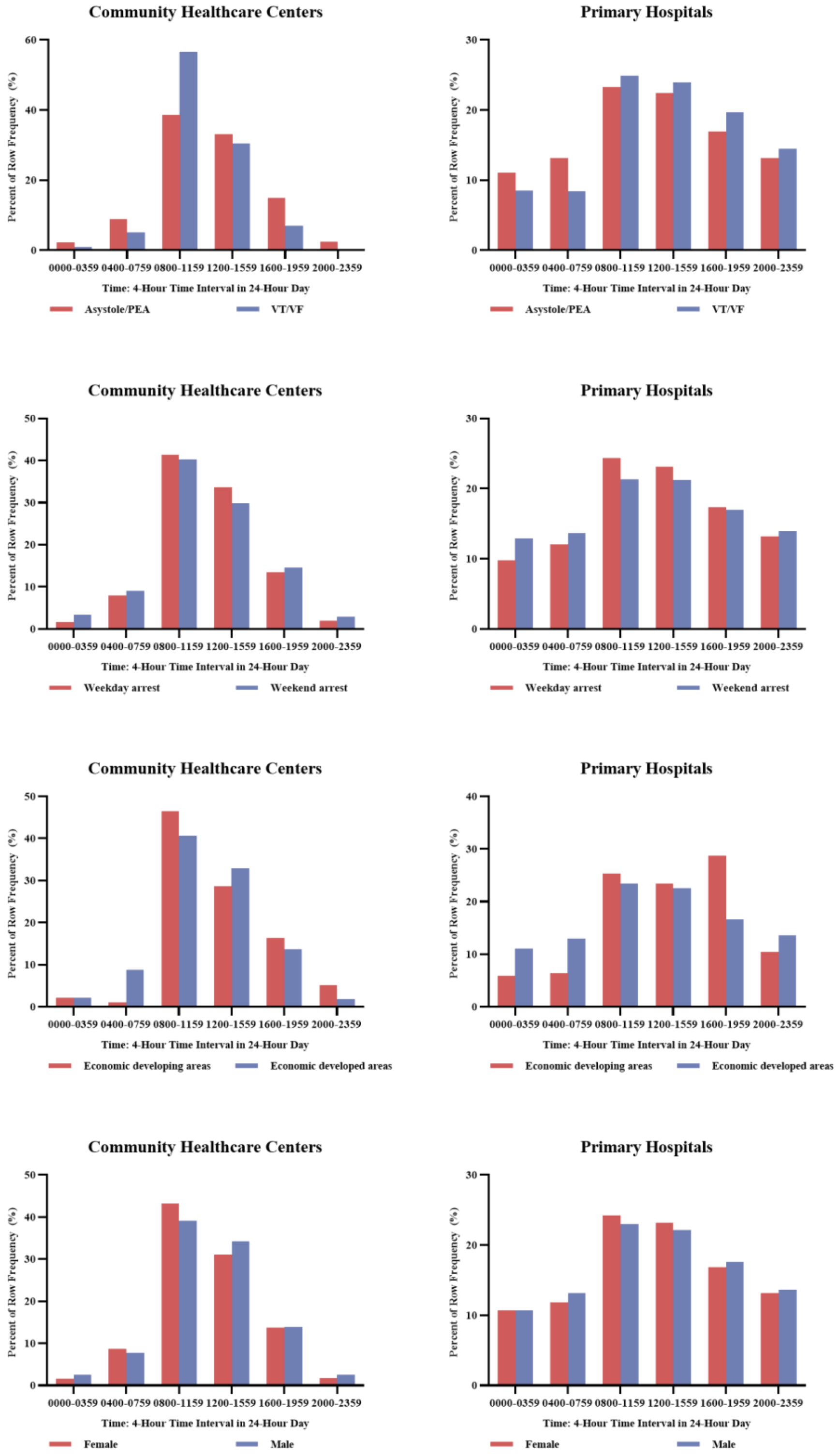

PEA = pulseless electrical activity; VF = ventricular fibrillation; VT = ventricular tachycardia.

Supplemental Figure 5. The weekly variation of sudden cardiac arrests in Chinese community.

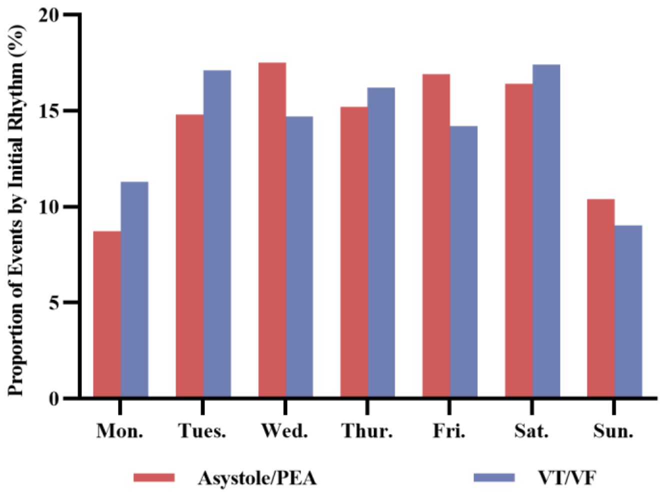

PEA = pulseless electrical activity; VF = ventricular fibrillation; VT = ventricular tachycardia.

Supplemental Figure 6. The seasonal variation of sudden cardiac arrests in Chinese community.

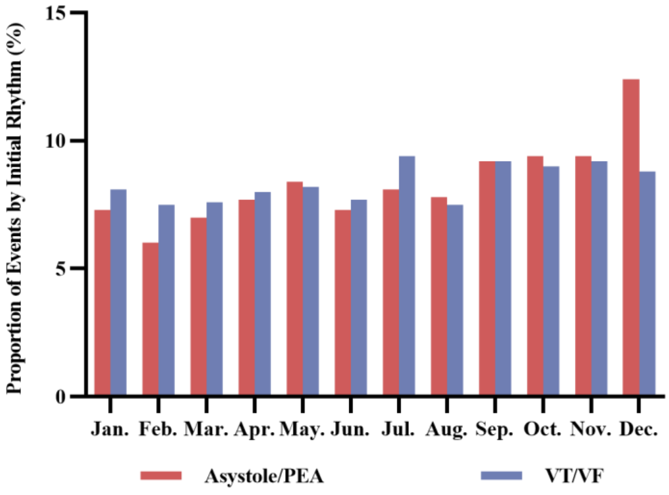

PEA = pulseless electrical activity; VF = ventricular fibrillation; VT = ventricular tachycardia.

Supplemental Table 1. Multivariable analysis of the risk of cardiac arrest due to primary hospitals compared to community healthcare centers.

Characteristics	Univariate		Multivariable	
	OR (95% CI)	P value	OR (95% CI)	P value
Time interval of arrest				
0000-0359	3.35 (2.57-4.37)	**<0.001**	3.64 (2.77-4.78)	**<0.001**
0400-0759	Reference		Reference	
0800-1159	0.38 (0.33-0.44)	**<0.001**	0.41 (0.35-0.48)	**<0.001**
1200-1559	0.46 (0.39-0.53)	**<0.001**	0.50 (0.42-0.58)	**<0.001**
1600-1959	0.83 (0.70-0.98)	**0.025**	0.87 (0.74-1.04)	0.125
2000-2359	4.05 (3.12-5.25)	**<0.001**	4.49 (3.43-5.87)	**<0.001**
Age	1.01 (1.00-1.01)	**<0.001**	1.01 (1.00-1.01)	**<0.001**
Male	1.23 (1.14-1.34)	**<0.001**	1.25 (1.14-1.36)	**<0.001**
Weekend arrest	1.14 (1.04-1.24)	**0.007**	1.06 (0.96-1.17)	0.289
Location of arrest				
East	0.61 (0.43-0.86)	**0.005**	0.47 (0.33-0.68)	**<0.001**
Central	0.03 (0.01-0.07)	**<0.001**	0.01 (0.00-0.03)	**<0.001**
Southeast	Reference		Reference	
Southwest	0.05 (0.03-0.08)	**<0.001**	0.00 (0.00-0.01)	**<0.001**
Northwest	119.76 (16.31-879.16)	**<0.001**	1.66 (0.07-38.60)	0.753
Economic developed areas	1.21 (1.03-1.43)	**0.019**	0.02 (0.00-0.18)	**0.001**
VT/VF (versus Asystole/PEA)	1.05 (0.93-1.17)	0.447		

CI = confidence intervals; OR = odds ratio; PEA = pulseless electrical activity; VF = ventricular fibrillation; VT = ventricular tachycardia.
